# Commencements

**Published:** 1898-08

**Authors:** 


### COMMENCEMENTS.

### Southern Medical College—Dental Department

The eleventh annual commencement exercises of the Dental Department of Southern Medical College were held in the Grand Opera House, Atlanta, Ga., on Saturday evening, April 2, 1898.

The annual oration was delivered by Professor George W. Macon, of Macon, Ga., and the valedictory by S. H. McAfee, D.D.S.

The number of matriculates for the session was eighty.

The degree of D.D.S. was conferred on the following graduates by Judge Howard Van Epps, President of the Board of Trustees:

| NAME. | RESIDENCE. | NAME. | RESIDENCE. |
|---|---|---|---|
| D Clement | Georgia | H R Parker | North Carolina |
| Guy C Estes | Florida | John O Pope | Alabama |
| Jesse K Floyd | Georgia | E E Roy | Wisconsin |
| D M Griffin | Florida | L A Smith | Georgia |
| J C Hill | Alabama | E M Wilder | Georgia |
| S H McAfee | Georgia | Alton Whitman | Florida |
| O G Pacetti | Cuba | | |

Total 13.

### New York College of Dentistry.

The thirty-second annual commencement exercises of the New York College of Dentistry were held in Chickering Hall, New York City, on Saturday evening, May 14, 1898.

The address to the graduates was delivered by Rev. J. Lewis Parks, D.D.

The degree of D.D.S. was conferred on the following graduates by Rev. George Alexander, D.D., President of the Board of Trustees:

| | | |
|---|---|---|
| William D Ayers | Rudolph F Hass | Bertram R Perkins |
| Joseph A Barrett | Jacob S Hyman | Thomas H Pratt |
| Charles Bokay | Alton A Jones | Joseph I Protassewitch |
| Julius M Bottstein | Ray M Kaulbach | Frank Quackenbush |
| Arthur R Bourne | James P Kelly | Thomas A Riordan |
| Frederick Boylhart | John T Kempton | Thomas J Seaman |
| Thomas F Clune | Albert Kerr | Joseph Sookne |
| Herbert J Cornell | Leo Konski | Julius Stern |
| Irving J Crump | Harold A Koonz | William Talbot |
| Wm St G Elliott, Jr | Frederick H Lum, Jr | John E Urich |
| Moise Garmidor | James Maddren | Edwin B Van Saun |
| Ray E Gilman | Thomas J Mangan | Albert F Van Winkle |
| Frank F Goodwin | Charles C Milligan | Thomas F Walsh |
| Abraham Greenberg | Raphael J Moolten | Reuben H Whitmyre |
| William H Gregory | William A Morris | John R Wyckoff |
| Charles C Halgren | Harry G Nolan | Arthur Young |

Total 48.

### Birmingham Dental College.

The annual commencement exercises of the Birmingham Dental College were held at O'Brien's Opera House, Birmingham, Ala., on Tuesday evening, April 5, 1898.

The annual address was delivered by Judge J. J. Banks, and the valedictory by H. T. Hamblen, D.D.S.

The number of matriculates for the session was thirty-four.

The degree of D.D.S. was conferred on the following graduates by T. M. Allen, D.D.S., dean:

| NAME. | RESIDENCE. | NAME. | RESIDENCE. |
|---|---|---|---|
| J W Balkcom | Alabama | H T Hamblen | Texas |
| W R Dillard | Mississippi | J W Kesy | Mississippi |
| I F Drummond | Alabama | H N McTyeire | Alabama |
| C F Ellis | Texas | R L Rogers | Georgia |

Total 8.

### Northwestern College of Dental Surgery.

The annual commencement exercises of the Northwestern College of Dental Surgery were held in the Auditorium, Chicago, Ills., on Thursday evening, April 7, 1898.

The salutatory address was delivered by L. A. Salisbury, and the valedictory by J. B. Curry, D.D.S.

The number of matriculates for the session was seventy-five.

The degree of D.D.S. was conferred on the following graduates by L. L. Davis, D.D.S., dean of the college and President of the Board of Trustees:

| NAME. | RESIDENCE. | NAME. | RESIDENCE. |
|---|---|---|---|
| W E Beil | Indiana | G Lindberg | Illinois |
| C W Betcher | Minnesota | H H Phillips | Michigan |
| H T Berry | Illinois | R Pruitt | Illinois |
| R R Bosworth | Illinois | L G Rand | Illinois |
| C W Bowbeer | Michigan | G R Riddell | Illinois |
| J B Curry | Wisconsin | L A Salisbury | Illinois |
| W G Dittmar | Illinois | Jas McC Shaffer | Illinois |
| E O Haese | Wisconsin | U Shantz | Canada |
| R R Hutchinson | Minnesota | J F Smith | Illinois |
| E X Jones | Illinois | E J Soik | Wisconsin |

Total 20.

### Tacoma College of Dental Surgery.

The annual commencement exercises of the Tacoma College of Dental Surgery were held at Chickering Hall, Tacoma, Washington, on Wednesday, April 6, 1898, at 8 o'clock P.M.

The closing announcement was delivered by J. M. Meyer, D.D.S., dean of the college; the valedictory by Geo. W. Stryker, D.D.S., and an address on the "Preliminary Education of Dental Students," by Professor J. L. Tait.

The number of matriculates for the session was thirty-three.

The degree of D.D.S. was conferred by J. W. Hickman, M.D., acting president of the college, upon the following members of the senior class:

| NAME. | RESIDENCE. | NAME. | RESIDENCE. |
|---|---|---|---|
| Francis Atwell | Idaho | Edward H Lennox | Washington |
| Henry D Brand | Washington | Frank E Morison | British Columbia |
| Andrew T Carlson | Washington | George W Stryker | Oregon |
| Clarence W Chamberlin | Washington | Elmer E Whittaker | Utah |
| Willard M King | Washington | | |

Total 9.

### Royal College of Dental Surgeons of Ontario.

On the 21st of April the directors of the Royal College of Dental Surgeons of Ontario handed out the result of the examinations. The number of students registered for the session of 1897–8 was 201, the number in attendance being 198.

The following passed the final examinations, and received the title of L.D.S.:

| NAME. | RESIDENCE. | NAME. | RESIDENCE. |
|---|---|---|---|
| A Almon Babcock | Ontario | John Hutchison | Ontario |
| Harvey J M Bannerman | Ontario | Harry Jackson | Ontario |
| John W Barker | Ontario | Geo G Jordan | Ontario |
| Donald H Beaton | Ontario | John A Lambertus | Ontario |
| George A Beattie | Ontario | Wm H Liddle | Ontario |
| Washington Buchanan | Ontario | J A Lotheed | Illinois |
| William H Bulmer | Ontario | Geo A Macoun | Ontario |
| Arthur C Burnet | Ontario | Samuel R Martin | Ontario |
| Mason J Clarke | Ontario | Stuart McL Milne | Ontario |
| Joseph Coghlan | Ontario | Frederick D McGrattan | Ontario |
| Chas A Cooke | Ontario | Thomas R Paterson | Ontario |
| Chas W Currie | Ontario | Samuel P Reynolds | Ontario |
| Arthur Day | Ontario | Andrew Scott | Ontario |
| Robt F Denike | Ontario | John Scott | Ontario |
| Adam R Donaldson | Ontario | Francis A Sellery | Ontario |
| Robert F Edmonds | Ontario | Wilber G L Spaulding | Ontario |
| Robert R Elliott | Ontario | Wm D Staples | Ontario |
| James B Gerry | Ontario | Thomas W Stoddart | Ontario |
| Frank W Glasgow | Ontario | James E Taggart | Ontario |
| Gordon Grant | Ontario | Wm F Taylor | Ontario |
| John W Hagey | Ontario | Adam H R Watson | Ontario |
| Robert R Harvie | Ontario | Freeman Waugh | Ontario |
| Wm J Hill | Ontario | Walter J Williams | Ontario |
| Jas A Hilliard | Ontario | Ogden A Winters | Ontario |
| Alfred E Hunt | Ontario | Wm H Woodrow | Ontario |

Total 50.

J. B. WILLMOTT, *Sec'y R. C. D. S.*

### Missouri Dental College.

The thirty-second annual commencement exercises of the Missouri Dental College (Dental Department of Washington University) were held at the Fourteenth Street Theater, St. Louis, Mo., on Thursday evening, April 28, 1898.

The number of matriculates for the session was 124.

The degree of Doctor of Dental Medicine was conferred by Chancellor Winfield S. Chaplin, of Washington University, upon the following graduates:

| NAME. | RESIDENCE. | NAME. | RESIDENCE. |
|---|---|---|---|
| Albert G Alexander | Missouri | Daniel M Kelsey | California |
| Burt Barry | Illinois | John P Leibrook | Illinois |
| Charles T Bedell | Missouri | George W Loesch | Missouri |
| Chas E Bellchamber | Missouri | Charles B Mitchell | Missouri |
| John C Boothe | Illinois | Edwin Moore | New York |
| Hermann Brandenberger | Missouri | Edwin D Morrow | Missouri |
| Ewing M Brite | Missouri | Ernest L Niemeyer | Illinois |
| Otis C Colby | Illinois | Benj T Owens | Texas |
| Charles C Crews | Virginia | Gilbert D Pearce | Missouri |
| Murton E DeGuire | Oregon | Karl P Pemberton | Montana |
| Roy H Ellis | Missouri | Fred B Rapp | Missouri |
| Alfred Fellner | Austria | Edw C Reisse, Ph.D | Missouri |
| George H Frank | Missouri | Noble G Rhodes | Missouri |
| Walter B Gorrett | Missouri | Huntington Sandel | Louisiana |
| Harry B Hammond | Missouri | Carl Schaer | Switzerland |
| Esco T Houston | Missouri | Rolla A Schwaner | Iowa |
| Quillen O Hudson | Missouri | George W Smith | Illinois |
| Charles J W Hugo | Missouri | James D Smith | Missouri |
| James W Hull | Missouri | Don Stocker | Missouri |
| Henry S Kimbrough | Missouri | | |

Total 39.

### National University—Dental Department.

The fourteenth annual commencement exercises of the Dental Department of the National University were held in the National Theater, Washington, D. C., on Wednesday evening, June 8, 1898.

The address to the graduating classes was delivered by Professor John T. Winter, M.D., and the valedictory by Seneca B. Bain, D.D.S.

The degree of D.D.S. was conferred on the following graduates by Hon. Richard H. Alvey, L.L.D., Chancellor of the University:

| NAME. | RESIDENCE. | NAME. | RESIDENCE. |
|---|---|---|---|
| P Bonnard Bain | Texas | Roue L Hogan | Nova Scotia |
| Seneca B Bain | Texas | Thos J McConnell | Indiana |
| Charles H Beach | Kentucky | Rosalind Moore | Dist. of Col. |
| Silas C Ellis | Missouri | Harry R Perry | Dist. of Col. |
| Chas H Fischer | New York | Chas W Radley | New York |
| Ernest W Fowler, M.D | Dist. of Col. | Jesse B Schafhirt | Dist. of Col. |
| Wm S Frankland | D st. of Col. | Edward S Smith | Dist. of Col. |
| Albert F Hodes | New York | Lena B Watson | Louisiana |

Total 16.

### University of Colorado—Dental Department.

The annual commencement exercises of the Dental Department of the University of Colorado were held at the St. James Hotel, Denver, Col., on Thursday evening, April 28, 1898.

The address to the graduates was delivered by Dr. Wm. T. Chambers, dean, and the valedictory by W. A. Brierley, D.D.S.

The degree of D.D.S. was conferred on the following graduates by Dr. H. A. Fynn, president of the college:

| NAME. | RESIDENCE. | NAME. | RESIDENCE. |
|---|---|---|---|
| Mahlon F Bauchert | Indiana | Henry Hoffman | Colorado |
| Mary R Bradner | Colorado | Ernest D Linton | Ohio |
| William A Brierley | Massachusetts | James L McMurray | New York |
| Robert Dunn | Colorado | Adam Turnbull | Scotland |
| Bellé R Hinrichs | California | George R Warner, Jr | Connecticut |

Total 10.

### Baltimore Medical College—Dental Department.

The third annual commencement exercises of the Dental Department of the Baltimore Medical College were held in

Ford's Opera House, Baltimore, Md., on Wednesday evening, April 6, 1898.

The oration was delivered by Rev. Curtis Lee Laws.

The degree of D.D.S. was conferred on the following graduates by S. K. Merrick, M.D., president of the college:

| NAME. | RESIDENCE. | NAME. | RESIDENCE. |
|---|---|---|---|
| William T Auer | Maryland | George M Maxwell | Maryland |
| Richard F Bartlett | Maryland | James M McFarland | Louisiana |
| Walter C Dorsey | Maryland | Peter McNichol | Canada |
| Justus H Ehlers | Maryland | William G Nixon | Virginia |
| Jesse D Gardner | North Carolina | Silas W Pope | New York |
| James F Gilbert | Rhode Island | Oren B Richards, Jr | Pennsylvania |
| William J Johnson | Canada | Franklin C Smith | Virginia |
| August M Marchant | Virginia | Hubert L Sprackling | Rhode Island |
| Louis H Marshall | Massachusetts | George G Yarrow | Massachusetts |

Total 18.

### New York Dental School.

The fifth annual commencement exercises of the New York Dental School were held in the Academy of Medicine, New York City, on Thursday, May 26, 1898.

The annual address was delivered by Rev. Joachim Elendorf, D.D.

The number of matriculates for the session was forty-seven.

The degree of D.D.S., from the University of the State of New York, was conferred on the following graduates by Dwight L. Hubbard, M.D., dean of the college:

| NAME. | RESIDENCE. | NAME. | RESIDENCE. |
|---|---|---|---|
| Frank Abel | New York | Wm C Rothkranz | New York |
| J V Andrews | New York | Wm Stack | New York |
| C W Bedford | New York | Carra Ulman | New York |
| Carl Klindt | New York | H D Westfehling | New York |
| Nils Odahl | Sweden | | |

Total 9.

### Central College of Dentistry.

The first commencement exercises of the Central College of Dentistry were held in the college building, Indianapolis, Ind., on Tuesday, April 11, 1898.

Addresses were delivered by Bishop White, of Indiana, and Dr. Joseph Eastman.

The number of matriculates for the session was twenty-four.

The degree of D.D.S. was conferred by Dr. Junius E. Cravens, president of the college, upon one graduate, William H. Matthews, of Indiana.

The college closed its first session with much satisfaction to the management and students.

### Washington Dental College.

The first annual commencement of the Washington Dental College was held Thursday evening, May 5, 1898, in the college building, Washington, D. C.

The address to the graduates was delivered by Professor W. Warrington Evans, M.D., D.D.S., president of the faculty.

The number of matriculates for the session was twenty-six.

The degree of D.D.S. was conferred on the following graduates: Wm. F. Baron, New Jersey, and Geo. C. Patton, Chicago, Ills.—Total 2.

### Howard University—Dental Department.

The annual commencement exercises of the Dental Department of Howard University were held in the Congregational Church, Washington, D. C., on Friday evening, May 6, 1898.

The annual address was delivered by J. E. Rankin, D.D., LL.D., president of the university, and the address to the graduates by Professor D. S. Lamb, A.M., M.D.

The number of matriculates for the session was twenty-four.

The degree of D.D.S. was conferred on the following graduates by President J. E. Rankin: Richard G. Baker, Pennsylvania; Raymond F. Crist and Clifton A. Johnson, Washington, D. C.; John E. Rattley, North Carolina; Riuzo Sohne, Japan. —Total 5.

### University of Denver—Dental Department.

The seventeenth annual commencement exercises of the Dental Department of the University of Denver were held in Trinity M. E. Church, Denver, Col., on Tuesday evening, May 10, 1898.

The address to the graduating class was delivered by Dr. A. H. Sawins, dean.

The number of matriculates for the session was forty-seven.

The degree of D.D.S. was conferred on the following graduates by Chancellor McDowell, of the university: Lucas H. E. Barnum, Kansas; Gen Rio Hara, California; Jay R. Haskin and Henry A. MacMillan, Colorado; Clarence S. Wilkinson, Illinois.—Total 5.

